# Phosphorylation-Dependent Regulation of Ca^2+^-Permeable AMPA Receptors During Hippocampal Synaptic Plasticity

**DOI:** 10.3389/fnsyn.2020.00008

**Published:** 2020-03-27

**Authors:** Alicia M. Purkey, Mark L. Dell’Acqua

**Affiliations:** Department of Pharmacology, University of Colorado School of Medicine, Anschutz Medical Campus, Aurora, CO, United States

**Keywords:** synaptic plasticity, LTP, LTD, Ca^2+^-permeable AMPA receptor, phosphorylation, PKA, calcineurin, AKAP

## Abstract

Experience-dependent learning and memory require multiple forms of plasticity at hippocampal and cortical synapses that are regulated by N-methyl-D-aspartate receptors (NMDA) and α-amino-3-hydroxy-5-methyl-4-isoxazolepropionic acid (AMPA)-type ionotropic glutamate receptors (NMDAR, AMPAR). These plasticity mechanisms include long-term potentiation (LTP) and depression (LTD), which are Hebbian input-specific mechanisms that rapidly increase or decrease AMPAR synaptic strength at specific inputs, and homeostatic plasticity that globally scales-up or -down AMPAR synaptic strength across many or even all inputs. Frequently, these changes in synaptic strength are also accompanied by a change in the subunit composition of AMPARs at the synapse due to the trafficking to and from the synapse of receptors lacking GluA2 subunits. These GluA2-lacking receptors are most often GluA1 homomeric receptors that exhibit higher single-channel conductance and are Ca^2+^-permeable (CP-AMPAR). This review article will focus on the role of protein phosphorylation in regulation of GluA1 CP-AMPAR recruitment and removal from hippocampal synapses during synaptic plasticity with an emphasis on the crucial role of local signaling by the cAMP-dependent protein kinase (PKA) and the Ca^2+^calmodulin-dependent protein phosphatase 2B/calcineurin (CaN) that is coordinated by the postsynaptic scaffold protein A-kinase anchoring protein 79/150 (AKAP79/150).

## Introduction

Long-term potentiation (LTP) and depression (LTD) can be induced by brief, strong vs. prolonged, weak activation of N-methyl-D-aspartate receptor (NMDAR) Ca^2+^ influx and are expressed by long-lasting increases or decreases, respectively, in α-amino-3-hydroxy-5-methyl-4-isoxazolepropionic acid receptor (AMPAR) activity. LTP/LTD at excitatory synapses can be induced rapidly (seconds-minutes) but expressed persistently (hours-days; Collingridge et al., 2010; Huganir and Nicoll, 2013). Hippocampal and cortical pyramidal neurons can also homeostatically scale-up or -down excitatory synaptic strength across all inputs in response to chronic (hours-days) decreases or increases, respectively, in overall input and firing (Turrigiano, 2012; Chen et al., 2013; Lee et al., 2013). Homeostatic synaptic plasticity, like Hebbian, is expressed through changes in AMPAR synaptic localization (O’Brien et al., 1998; Turrigiano et al., 1998; Thiagarajan et al., 2005; Sutton et al., 2006; Aoto et al., 2008; Ibata et al., 2008; Lee and Chung, 2014). However, it was originally thought that the mechanisms mediating Hebbian and homeostatic AMPAR regulation would not be identical due to several opposing features. For instance, LTP and homeostatic scaling-up are triggered by brief, elevated vs. prolonged, decreased Ca^2+^ signaling. Nonetheless, accumulating evidence indicates that the mechanistic lines separating Hebbian and homeostatic plasticity are becoming blurred with common signaling machinery controlling both processes. Importantly, Hebbian and homeostatic synaptic plasticity alterations are implicated in many nervous system disorders, including Alzheimer’s disease, Fragile X, Rett syndrome, and autism, thus, we need to understand the underlying signaling mechanisms (Thiagarajan et al., 2007; Keck et al., 2017). This review article will briefly review the respective roles of NMDARs and AMPARs in synaptic transmission but will primarily focus on mechanisms regulating the activity, trafficking, and subunit composition of synaptic AMPARs during synaptic plasticity, with emphasis on CP-AMPAR regulation in CA1 hippocampal pyramidal neurons.

## Ionotropic Glutamate Receptors and the Postsynaptic Density (PSD)

Glutamatergic synapses on principal cells in the CNS, such as hippocampal and cortical pyramidal neurons, are predominately located on dendritic spines and contain a structure known as the postsynaptic density (PSD), so named based on its appearance in electron micrographs due to the densely-packed protein network it contains (Sheng and Hoogenraad, 2007). In the 1970s, the first PSD purification experiments were carried out and in the 1990s the first molecular constituents of the PSD components were identified. Owing largely to the development of mass spectrometry-based proteomics, many PSD proteins have been identified in the past few decades. The average PSD has a molecular mass of ~1 gigadalton (Chen et al., 2005) and contains 100–1,000 different proteins, including most prominently NMDARs and AMPARs, scaffolding proteins, voltage-gated ion channels, cell adhesion molecules, cytoskeletal elements and intracellular signaling enzymes. One of the most abundant and first identified components of the PSD is postsynaptic density protein 95 (PSD-95; Cho et al., 1992), which is the most prominent member of a family of PDZ-domain-containing membrane-associated guanylate kinase (MAGUK) scaffold proteins that serve as primary organizers of PSD structure and master regulators of excitatory synapse function (Won et al., 2017). Despite its complex composition, the PSD is a dynamic structure with changes in protein composition taking place in hours-days over the course of synaptic development and homeostatic plasticity and in seconds-minutes following the induction LTP or LTD. While we now appreciate a whole host of molecular players within the PSD, we still do not have a thorough understanding of the molecular organization of the PSD or how its protein composition and those of the associated synaptic membrane plus neighboring perisynaptic (within 100 nm of the PSD) and extrasynaptic regions of the dendritic spine plasma membrane are regulated during plasticity (Sheng and Hoogenraad, 2007).

**Figure 1:**
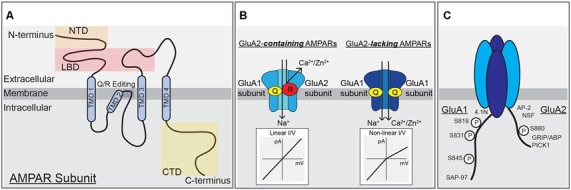
α-amino-3-hydroxy-5-methyl-4-isoxazolepropionic acid (AMPA) receptor subunit structure, function and modifications. **(A)** A single AMPAR subunit with (N)-terminal domain (NTD), ligand-binding domain (LBD), transmembrane domain (TMD), and (C)-terminal domain (CTD) structural domains indicated. **(B)** AMPARs containing the GluA2-subunit are unable to pass calcium due to the positive charge of arginine residues within the pore, *left*. AMPARs lacking the GluA2-subunit can pass calcium and have a non-linear, inwardly rectifying current-voltage relationship due to block of outward current by intracellular polyamines, *right*. **(C)** Schematic of the CTDs of GluA1 and GluA2 highlighting phosphorylation sites and protein-protein interaction domains.

Ionotropic glutamate receptors are the major functional component of the PSD that mediate excitatory synaptic transmission. These receptors are integral membrane proteins that form ion channels from four individual subunits coming together to form tetrameric receptors with cation-selective pores (Traynelis et al., 2010). Each subunit is composed of four domains: an amino (N)-terminal domain (NTD) that drives multimerization, a highly conserved extracellular clamshell-like ligand-binding domain (LBD), which together comprise ~85% of receptor mass and protrude ~130 Angstroms into the synaptic cleft (Sobolevsky et al., 2009; Meyerson et al., 2014; García-Nafría et al., 2016), the transmembrane domain (TMD) containing the ion-conducting pore, and a variable intracellular carboxy (C)-terminal domain (CTD; Figure 1A). There are three major classes of ionotropic glutamate receptors that mediate synaptic transmission at cortical and hippocampal synapses: AMPA receptors, kainate receptors (KARs), and NMDA receptors. AMPARs, KARs, and NMDARs are all activated by glutamate binding to their LBDs but with NMDARs also requiring binding of glycine or D-serine as a co-agonist. Upon agonist binding the LBDs change conformation causing the ion channel pore in the TMD to open and allow Na^+^, K^+^, and in some cases Ca^2+^ and Zn^2+^ cation flux (Traynelis et al., 2010). The forms of hippocampal synaptic plasticity covered in this review are only regulated by AMPARs and NMDARs, thus KARs will not be further discussed.

## NMDA Receptors

NMDARs form the functional core of the synapse with ~20 NMDARs per PSD (Sheng and Hoogenraad, 2007). Unlike AMPARs that are highly variable in number from spine to spine, the number of NMDARs is fairly consistent across synapses and in general is more stable over time (Sheng and Hoogenraad, 2007). NMDARs are heterotetramers formed by two GluN1 subunits (*Grin1* gene) that bind the co-agonists glycine and D-serine and two-variable GluN2 or GluN3 subunits that bind glutamate or glycine, respectively (Traynelis et al., 2010; Gray et al., 2011). NMDAR subunit expression is variable throughout the brain across different cell types and during development and can contribute to differences in NMDAR channel properties, including desensitization and Ca^2+^-conductance. The majority of NMDARs in hippocampal CA1 neurons contain GluN1 in various combinations with GluN2A (*Grin2A* gene) and GluN2B (*Grin2b* gene) subunits (Traynelis et al., 2010). While AMPARs are purely ligand-gated, NMDARs are not only directly ligand-gated but are also indirectly voltage-gated by virtue of the requirement for membrane depolarization to relieve pore block by Mg^2+^ ions. As a result of this voltage-dependent Mg^2+^ pore block, NMDARs are not responsible for much of the current at the resting membrane potential of −70 mV during basal transmission, but when activated in response to repetitive stimuli that induce synaptic plasticity, glutamate binding coincident with postsynaptic depolarization mediated by AMPAR activation allows the NMDAR to open and conduct Na^+^ and Ca^2+^ inward and K^+^ outward. While NMDAR Ca^2+^-current makes up only a small percentage of the total current passed through the channel, it is essential for neuronal signaling that regulates AMPAR activity in synaptic plasticity.

## AMPA Receptors

AMPARs are the primary mediators of fast excitatory glutamatergic neurotransmission in the CNS under basal conditions. Due to their rapid kinetics, opening and closing on the timescale of milliseconds, AMPARs allow for fast depolarization of the postsynaptic membrane *via* Na^+^ influx and thus high-fidelity propagation of signaling between pre- and postsynaptic neurons. AMPARs form tetramers of homo- and heterodimers composed of GluA1–4 subunits (genes *Gria1–4*), and are, like NMDARs, dimers of dimers (Lu et al., 2009; Traynelis et al., 2010). Channel opening depends on glutamate binding to all subunits of the tetramer (Lisman et al., 2007). GluA1–4 subunits can contribute differently to receptor properties like channel kinetics, ion selectivity, and intracellular trafficking. In addition to innate subunit-specific properties, mRNA processing, auxiliary proteins and phosphorylation add additional complexity to subunit control of receptor properties. AMPAR GluA1–4 subunits differ the most from each other in their divergent CTDs that vary in length and serve as a major site for regulatory intracellular protein-protein interactions and post-translational modifications (Figures 1A,C; Shepherd and Huganir, 2007; Traynelis et al., 2010; Benke and Traynelis, 2019).

AMPAR synaptic number varies widely from synapse to synapse reflecting differences in synaptic strength (Sheng and Hoogenraad, 2007). Using super-resolution imaging techniques, individual hippocampal synapses are thought to contain 20–100 AMPARs organized into distinct nanoclusters containing on the order of 20–40 receptors (Biederer et al., 2017; Chen et al., 2018; Choquet, 2018). AMPARs are highly mobile and their synaptic abundance is highly regulated developmentally and during synaptic plasticity. Much work has gone into understanding AMPAR trafficking to and from synapses to control synaptic strength and how receptor subunit composition can influence AMPAR properties.

### Ca^2+^-Permeable AMPA Receptors

AMPAR channel function is prominently controlled by the presence or absence of the GluA2 subunit. Interestingly, the impacts of GluA2 on AMPAR function are a product of adenosine deaminase mediated post-transcriptional editing of the *Gria2* mRNA that precedes mRNA splicing and translation. This mRNA-editing occurs at codon 607 and the resulting residue of the GluA2 protein is located in the membrane re-entrant pore loop (Figures 1A,B). Editing at this position results in a Glutamine to Arginine (Q/R) substitution that reduces overall channel conductance, limits permeability to Ca^2+^ (and Zn^2+^), and prevents pore block by positively charged polyamines, all due to the introduction of two large positively charged R residues in the pore. The introduction of R residues into the pore of GluA2-containing AMPARs also influences receptor assembly in endoplasmic reticulum (ER) to favor heterodimerization with other subunits and ER exit over homodimerization to form GluA2-homomers that are retained in ER and if they reached the surface would have very little activity (Greger et al., 2003; Traynelis et al., 2010). However, the process of AMPAR dimer assembly itself is driven by interactions between the NTDs, and recently GluA1 NTD interactions have been shown to be key for regulating synaptic incorporation (Díaz-Alonso et al., 2017; Watson et al., 2017). As the mRNA editing process is normally very efficient, most GluA2 subunits are Q/R edited, resulting in low Ca^2+^-permeability and insensitivity to polyamine blockade (Ca^2+^-impermeable AMPARs, CI-AMPARs). Alternatively, AMPAR assemblies lacking GluA2 subunits, such as GluA1 homomers, are Ca^2+^-permeable (i.e., CP-AMPARs), though still less so than NMDARs (Isaac et al., 2007; Traynelis et al., 2010). CP-AMPARs are sensitive to channel block by endogenous intracellular polyamines, such as spermine, and exogenously applied extracellular polyamine toxins and compounds, such as philanthotoxin (PhTx), joro spider toxin, argiotoxin, IEM-1460, and 1-naphthylacetyl-spermine (NASPM; Blaschke et al., 1993; Herlitze et al., 1993; Bowie and Mayer, 1995; Koike et al., 1997; Magazanik et al., 1997; Washburn et al., 1997; Toth and McBain, 1998). These exogenous polyamine-derivatives can be extracellularly applied to produce open-channel block of CP-AMPARs, and are thus frequently used to probe receptor subunit composition in neurons (Toth and McBain, 1998; Liu and Cull-Candy, 2000; Kumar et al., 2002; Terashima et al., 2004; Plant et al., 2006).

In addition, CI-AMPARs and CP-AMPARs display different current-voltage (I–V) relationships due to block of CP-AMPARs by intracellular polyamines at positive potentials. All AMPARs, like NMDARs, have a reversal potential near 0 mV due to lack of selectivity for Na^+^ vs. K^+^, but while GluA2-containing CI-AMPARs exhibit a linear I-V relationship at potentials both negative and positive to 0 mV, GluA2-lacking CP-AMPARs exhibit very little current at membrane potentials greater than 0 mV due to endogenous polyamines being driven into the pore in a voltage-dependent manner and preventing outward flux of K^+^ ions. This phenomenon of passing less outward current than inward current is called inward rectification (Figure 1B). As mentioned above, the presence of GluA2 also regulates AMPAR single-channel conductance, with GluA1 homomers conducting an average of ~12 pS and GluA1/2 heteromers passing much less current at ~3 pS (Benke and Traynelis, 2019). From numerous studies it appears the majority of AMPARs under basal conditions at most synapses on most principal cells in the brain, including in CA1 hippocampal pyramidal cells (Lu et al., 2009), are heteromeric GluA2-containing CI-AMPARs with low single-channel conductance. However, under certain conditions, both physiological and pathophysiological, a small number of GluA2-lacking CP-AMPARs with high single-channel conductance can be recruited to synapses to play a critical role in modifying synaptic signaling during plasticity and disease (Cull-Candy et al., 2006; Liu and Zukin, 2007; Man, 2011). In cortical and CA1 pyramidal cells, these CP-AMPARs are mainly thought to be GluA1 homomers, except very early in development when GluA4 is more abundantly expressed (Zhu et al., 2000).

### AMPAR Variable CTD Contributions to Subunit Regulation

Because AMPAR subunits are otherwise highly homologous, the variable CTD is thought to be a site of conferring distinct modes of regulation between the subunits, including membrane trafficking, stabilization, and degradation. GluA1 and GluA4 have long CTDs and GluA2 and GluA3 have short CTDs that contain a number of sites for subunit-specific post-translational modification, including phosphorylation, and protein-protein interactions, such as with different scaffold proteins and cytoskeletal elements (Henley et al., 2011; Figure 1C). Initially, the NMDAR GluN2A and GluN2B CTDs were identified as directly binding to the PDZ domain-containing MAGUK scaffold protein PSD-95 (Sheng and Kim, 2011) and the AMPAR GluA1 CTD as directly binding to the related MAGUK Synapse-associated protein 97 (SAP97; Leonard et al., 1998). This MAGUK family of PDZ scaffolds also includes PSD-93 and SAP102, with functions of these four MAGUKs having some overlap (Xu, 2011; Zheng et al., 2011). Overall, the expression of MAGUKs, PSD-95 in particular, is important for maintaining both AMPAR and NMDAR targeting to the synapse (Chen et al., 2015). Accordingly, PSD-95 indirectly interacts with AMPARs independent of subunit composition through PDZ binding to the C-terminal tail of the auxiliary transmembrane AMPA receptor regulatory proteins (TARPs), which both modify channel biophysical properties and promote AMPAR retention at synapses (Straub and Tomita, 2012).

**Figure 2:**
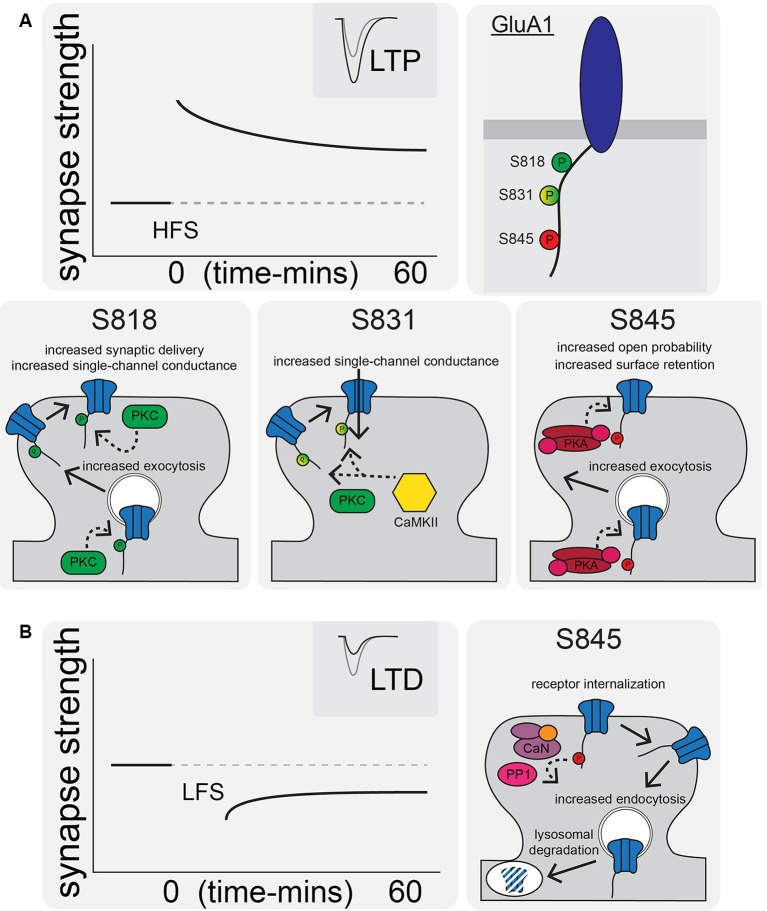
AMPAR synaptic trafficking regulation by CTD phosphorylation during long-term potentiation (LTP) and depression (LTD). **(A)** LTP stimuli induce phosphorylation at S818, S831, and S845 on the GluA1 CTD. Phosphorylation of these sites by CaMKII, PKC, and/or PKA increases synaptic AMPAR content and increases receptor transmission by a variety of indicated mechanisms. **(B)** LTD is characterized by AMPAR internalization and increased lysosomal degradation *via* CaN- and protein phosphatase 1 (PP1)-mediated dephosphorylation of GluA1 S845.

CTD phosphorylation of different AMPAR subunits can regulate channel properties and localization. GluA1–4 subunits are phosphorylated at over 20 serine, threonine, and tyrosine residues by many kinases, such as Calcium/calmodulin-dependent protein kinase II (CaMKII), PKA, Protein Kinase C (PKC), Protein Kinase G (PKG), proto-oncogene tyrosine-protein kinases Src and Fyn, and c-Jun N-terminal kinase (JNK; Shepherd and Huganir, 2007; Lu and Roche, 2012). In particular, GluA1 CTD phosphorylation has been extensively studied with three sites prominently featured in control of receptor activity and trafficking: Serine 818 (S818), Serine 831 (S831), and Serine 845 (S845; Figures 1C, 2A). Phosphorylation of S818 by PKC both increases single-channel conductance and promotes GluA1 surface delivery and synaptic incorporation (Figure 2A; Diering and Huganir, 2018). CaMKII and PKC phosphorylate S831, which can increase single-channel conductance and control receptor trafficking and synaptic incorporation (Figure 2A; Diering and Huganir, 2018; Summers et al., 2019). GluA1 S845 is phosphorylated by PKA and PKG and is involved in both regulation of open probability (Banke et al., 2000) and receptor recycling between intracellular endosomes and the extrasynaptic plasma membrane (Figures 2A,B; Traynelis et al., 2010). In particular, S845 phosphorylation appears to promote endosomal recycling of GluA1 containing receptors, including GluA1 homomeric CP-AMPARs, to prevent their sorting to late endosomes and the lysosome for degradation (He et al., 2009; Fernández-Monreal et al., 2012), and to promote GluA1 delivery to the extrasynaptic membrane (Sun et al., 2005; Oh et al., 2006; Man et al., 2007; Yang et al., 2008, 2010; He et al., 2009). It has been determined that ~15% of receptors are phosphorylated at S831 and S845 at rest (Diering et al., 2016; but see also Hosokawa et al., 2015). As detailed more below, these phosphorylation events appear to play a critical role in controlling receptor trafficking and function during LTP, LTD and homeostatic synaptic plasticity.

GluA2 trafficking and synaptic localization are also regulated by phosphorylation and protein-protein interactions with its CTD. In the 1990s, yeast two-hybrid screens identified a number of proteins that interact with the GluA2 CTD, including the PDZ interactions between GluA2 (and GluA3) and GRIP 1 and 2 [Glutamate Receptor Interacting Protein (GRIP1)/AMPAR Binding Protein (ABP)] and Protein Interacting with C Kinase (PICK1; Figure 1C; Dong et al., 1997, 1999; Lüscher et al., 1999; Srivastava and Ziff, 1999; Dev et al., 2000; Xia et al., 2000). In addition, both N-ethylamine-Sensitive Factor (NSF), a protein required for membrane fusion and exocytosis, and AP2, a protein required for clathrin-dependent endocytosis, interact with the juxtamembrane region of GluA2 CTD. Accordingly, the GluA2-NSF interaction is important in maintaining AMPAR content at the synapse, while the AP2 motif mediates endocytic removal (Nishimune et al., 1998; Osten et al., 1998; Song et al., 1998; Lüscher et al., 1999; Lüthi et al., 1999; Noel et al., 1999; Lee et al., 2002). The GluA2 subunit CTD can also be modulated by phosphorylation of Y875 by Src, which is then dephosphorylated to favor endocytosis during LTD. Phosphorylation of Serine 880 within the PDZ ligand domain by PKC (Figure 1C) disrupts GluA2 binding to GRIP1/2 but increases binding to PICK1 to promote trafficking in both directions between the plasma membrane and endosomes (Matsuda et al., 1999; Chung et al., 2000; Gladding et al., 2009; Collingridge et al., 2010).

## AMPAR Regulation During LTP and LTD

**Figure 3:**
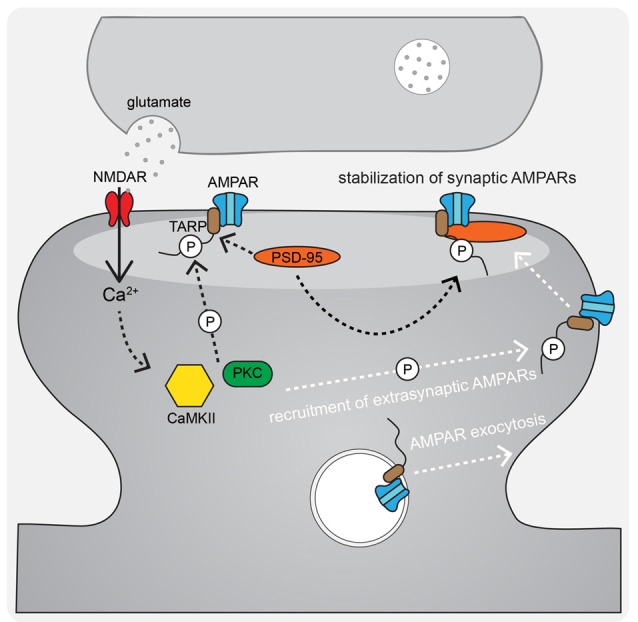
AMPAR-TARP interaction and TARP phosphorylation regulate AMPAR diffusional trapping in the postsynaptic density (PSD) during LTP. During LTP stimuli, N-methyl-D-aspartate receptors (NMDAR)-Ca^2+^ activated CaMKII and PKC phosphorylate AMPAR-associated TARPs both to trap newly exocytosed extrasynaptic receptors in the synapse after lateral diffusion and to stabilize existing synaptic receptors by binding to the synaptic scaffold PSD-95.

During LTP induction, AMPARs are activated and relieve NMDAR pore blockade by Mg^2+^ to permit Ca^2+^ entry into the postsynaptic cell and initiate signaling cascades that result in changes in synaptic strength (Kessels and Malinow, 2009; Huganir and Nicoll, 2013). The postsynaptic mechanisms required for LTP downstream of NMDAR-Ca^2+^ include, most prominently, signaling by the protein kinases CaMKII (*via* Ca^2+^-calmodulin), PKA (*via* Ca^2+^-sensitive adenylyl cyclase-mediated cAMP production), and PKC (*via* Ca^2+^ and phospholipase C lipid signaling). In particular, CaMKII activity is necessary and can even be sufficient for mediating LTP induction and expression (Nicoll, 2017). AMPAR regulation downstream of these kinase signaling cascades involves changes in phosphorylation state of the AMPARs themselves (Figure 2A) as well as the auxiliary TARP proteins (Figure 3) to control both channel biophysical properties and synaptic receptor number *via* endo- and exocytosis and lateral diffusion and synaptic insertion (Shepherd and Huganir, 2007; Newpher and Ehlers, 2008; Opazo and Choquet, 2011; Huganir and Nicoll, 2013; Buonarati et al., 2019).

### CP-AMPARs in LTP and LTD

Although early studies found mainly a role for AMPARs in LTP expression and no requirement in LTP induction, beyond facilitating relief of NMDAR Mg^2+^ block (Kauer et al., 1988; Muller et al., 1988), considerable research has since indicated that AMPARs can play more active roles in controlling both plasticity induction and expression in the hippocampus and other brain regions. While it has long been appreciated that NMDARs are required for induction of LTP at CA1 synapses and that the Ca^2+^ they provide is an important signal for LTP (the NMDAR competitive antagonist AP5 and open channel blocker MK801 both prevent induction of LTP), more recent studies (Plant et al., 2006; Lu et al., 2007; Guire et al., 2008; Yang et al., 2010; Sanderson et al., 2016) have implicated another Ca^2+^ source, the CP-AMPAR, as an additional key regulator of LTP, as well as LTD (but see also Adesnik and Nicoll, 2007; Gray et al., 2007). While GluA2-lacking, GluA1 homomeric CP-AMPARs are largely excluded from hippocampal synapses basally (Lu et al., 2009; Rozov et al., 2012), both Hebbian and homeostatic plasticity can modify synaptic strength *via* recruiting CP-AMPARs to synapses (Thiagarajan et al., 2005; Plant et al., 2006; Sutton et al., 2006; Lu et al., 2007; Aoto et al., 2008; Yang et al., 2010; Soares et al., 2013; Park et al., 2016; Sanderson et al., 2016; but see Adesnik and Nicoll, 2007; Ancona Esselmann et al., 2017). These recruited CP-AMPARs, due to both greater single-channel conductance and Ca^2+^ permeability described above, can in turn not only influence the level of plasticity expression but also confer changes in synaptic signaling resulting in the plasticity of plasticity i.e., metaplasticity. Importantly, CP-AMPAR metaplasticity in the VTA, nucleus accumbens and amygdala has been linked to drug addiction and fear extinction (Clem and Huganir, 2010; Wolf and Tseng, 2012). However, the roles of CP-AMPARs in plasticity and metaplasticity in the cortex and at CA1 synapses in the hippocampus remain controversial, in large part because we do not have an adequate understanding of the mechanisms that determine whether CP-AMPARs are recruited to or removed from synapses.

CP-AMPARs, as identified both by their inward rectification and sensitivity to polyamine-derived drugs [such as NASPM, IEM, and PhTx (Traynelis et al., 2010)], have been found to be transiently recruited to synapses in CA1 pyramidal neurons in response to induction of both LTP and LTD (Plant et al., 2006; Lu et al., 2007; Guire et al., 2008; Yang et al., 2010; Jaafari et al., 2012; Park et al., 2016; Sanderson et al., 2016). These recruited CP-AMPARs are then subsequently removed within ~15–30 min of the LTP induction stimulus (Plant et al., 2006) or during the prolonged (6–15 min) LTD induction stimulus (Sanderson et al., 2016). Accordingly, blocking CP-AMPARs with antagonists at early time points after LTP induction will prevent LTP, but not at later time points ~30 min after induction, when LTP expression is fully established (Washburn and Dingledine, 1996; Plant et al., 2006; Yang et al., 2010; Jaafari et al., 2012). These observations indicate that CP-AMPARs are important in a short window following induction and that early Ca^2+^ entry through these receptors can be important for establishing the stable expression of LTP but not in maintaining LTP expression once fully established. Likewise, CP-AMPAR antagonists reduce the amount of resulting LTD expression only when applied during LTD induction, when they are present, but not later after induction of LTD, when expression is established and the previously recruited CP-AMPARs have already been removed (Sanderson et al., 2016). Because there are few or no synaptic CP-AMPARs basally, transient introduction of a very small number of these high conductance receptors can have a large impact on CA1 synaptic strength; only a ~5% increase in synaptic CP-AMPAR content is needed to account for the increased conductance seen during a typical LTP experiment (Guire et al., 2008; Stubblefield and Benke, 2010; Benke and Traynelis, 2019). Hence, an attractive and experimentally supported model is that CP-AMPARs help to increase postsynaptic currents for a short yet critical period after LTP induction or during LTD induction to promote additional Ca^2+^ signaling that is required for promoting stable expression in the case of LTP or maximal induction in the case of LTD. However, it not yet known what specific downstream signaling pathways this additional CP-AMPAR synaptic Ca^2+^ influx is engaging to promote LTP vs. LTD.

### Controversy Surrounding CP-AMPAR Involvement in LTP

Although multiple lines of investigation suggest that CP-AMPARs can be recruited during LTP, significant controversy still exists due to other studies showing there is no GluA1 homomer involvement (Adesnik and Nicoll, 2007; Gray et al., 2007; Granger et al., 2013). It has become clear over time and with more experimental evidence that a number of variables could be contributing to these inconsistencies regarding CP-AMPAR involvement in LTP, including most prominently developmental age of the animals (which also makes it difficult to directly compare between rats and mice), the recording methods (extracellular vs. whole-cell), and the induction protocols used (Table 1). Our laboratory and others found that in mice at ~P14 (2 weeks of age), robust recruitment of CP-AMPARs can be observed following 1 × 100 Hz induction of LTP using extracellular field recording, but this CP-AMPAR recruitment by 1 × 100 Hz LTP disappears by between ~P17–21 and then reappears at ages >P42 (Lu et al., 2007; Sanderson et al., 2016). However, another variable impacting CP-AMPAR involvement in LTP is the specific type of plasticity being induced. Not only has it emerged that there exist many types of plasticity *in vivo* (Lisman, 2017), but also within the literature there exist many diverse protocols for inducing LTP *ex vivo* in brain slices, whether using an extracellular or whole-cell recording. The choice of induction protocol likely plays a pivotal role in the signaling pathways initiated and how they interact with the mechanisms that recruit CP-AMPARs. In general, it appears that CP-AMPAR recruitment after LTP is more likely to be observed when induced using relatively brief, weak stimuli (1–2× HFS tetani, single or spaced theta-burst stimulation (sTBS), briefer 0 mV pairing) compared to stronger stimuli (multiple HFS tetani, massed/continuous theta burst (cTBS), prolonged 0 mV pairing), such that at even a single developmental age one can observe both CP-AMPAR dependent and independent forms of LTP depending on the induction protocol. For example, we found using whole-cell recording in ~2–3 week-old mice that a relatively weaker, brief 2 × 100 Hz, 1 s HFS, 0 mV pairing induction protocol resulted in moderate LTP expression that was sensitive to the CP-AMPAR blocker NASPM, while a stronger, prolonged 3 Hz, 90 s 0 mV pairing induction protocol resulted in more robust LTP expression that was insensitive to NASPM (Purkey et al., 2018). In addition, work from Guire et al. (2008) found using extracellular field recording from 4 to 6 week-old rats that a weaker, brief TBS induction stimulus-induced LTP was CP-AMPAR dependent, while a stronger 3 × 100 Hz HFS induction stimulus-induced LTP was CP-AMPAR independent.

**Table 1:** Ca^2+^-permeable α-amino-3-hydroxy-5-methyl-4-isoxazolepropionic acid receptors (CP-AMPAR) plasticity studies.

References	Age/Species	LTP induction protocol	CP-AMPAR? (*no*; *yes*)
Gray et al. (2007)	2 (P15–17)-3 (P21–23) weeks, 8–12 weeks, Mouse	Fields: 2 × 100 Hz, 10 s interval; Whole-cell: 2 Hz, 100 pulses paired −10 mV holding; current-clamp recordings	All were insensitive to 100–200 μM IEM1460.
Adesnik and Nicoll (2007)	2–3 weeks, Mouse and Rat	Fields: 4 × 100 Hz, 20 s interval; Whole-cell: 2 Hz, 120 pulses paired between −10 to 0 mV	All insensitive to 10 μM PhTx-433. No rectification changes observed any time after LTP induction.
Granger et al. (2013)	P17–20, Mouse	Whole-cell: 2 Hz, 90 s at 0 mV	*Gria1–3^fl/fl^*; rescued with mutant receptors (all were Ca^2+^ permeable). No single AMPAR subunit found to be important for LTP.
Plant et al. (2006)	2–3 weeks, Mouse	Whole-cell: 0.5–2 Hz, 50–100 pulses paired to 0 or −10 mV	Rectification changes observed for ~15–30 min post-induction; sensitive to 10 μM PhTx-433.
Guire et al. (2008)	4–6 weeks, Rat	Fields: TBS (five trains at 5 Hz, four pulses at 100 Hz per train) or HFS (3 × 100 Hz, 1 s, 20 s interval)	TBS stim (not HFS) sensitive to 30 μM IEM1460 immediately after induction (not 20 min later).
Lu et al. (2007)	2 (P12–14), 3 (P20–22), 4, 8 weeks, Mouse	Fields: 1 × 100 Hz, 1 s	1 × 100 Hz: 2 and 8 week-old sensitive to 2.5 μM PhTx and 20 μM NASPM; 3 and 4 week-old insensitive.
		2 × 100 Hz, 1 s spaced 20 s	2 × 100 Hz: insensitive.
Yang et al. (2010)	P13–18, Rat	Fields: TBS (three trains at 5 Hz, five pulses at 100 Hz per train, 2 ×, 20 s interval)	Incomplete expression of LTP with 10 μM PhTx-433, Ca^2+^ entry from CP-AMPARs required for LTP.
Sanderson et al. (2016)	2 (P11–14), 3 (P17–21) weeks, Mouse	Fields: LTP 1 × 100 Hz, 1 s; LTD 1 Hz, 15 min; Whole-cell: LTD 1 Hz, 6 min paired at −30 mV.	2 week-old LTP 70 μM IEM1460 sensitive when added immediately post-induction, 3 week-old LTP insensitive. 2 week-old LTD sensitive to 30 μM NASPM added during induction. Rectification changes observed transiently during LTD induction but not after.
Park et al. (2016)	3–12 weeks, Rat	Fields: cTBS 3 TBS episodes, 10 s interval; sTBS 3 TBS episodes, 2 min-1 h interval; wTBS 1 TBS episode	wTBS, cTBS insensitive to 30 μM IEM1460; sTBS sensitive to 30 μM IEM1460.
Zhou et al. (2018)	3–4 weeks, Mouse	Whole-cell: LTP 1 × 100 Hz, 4 × 100 Hz; Fields: 100 Hz, 1 s 1 or 4 times with inter-train interval of 10 s or 5 min	LTP depends on GluA1 C- tail; did not address CP-AMPARs but may be involved - GluA1 requirement and conductance change.
Purkey et al. (2018)	2–3 weeks, (P14-P21) Mouse	Whole-cell: LTP 2 × 100 Hz at 0 mV, 3 Hz, 90 s at 0 mV	Weaker 2 × 100 Hz LTP sensitive to NASPM but stronger 3 Hz, 90 s LTP insensitive to NASPM.

However, what constitutes a weak vs. strong LTP induction protocol may be different between whole-cell and extracellular recording approaches; for instance, in mice at ~2 weeks of age, we found that 2 × 100 Hz HFS with 0 mV pairing induces CP-AMPAR dependent LTP while others using 2 × 100 Hz induction in extracellular field recording found LTP at this same age that was insensitive to CP-AMPAR blockers (Gray et al., 2007; Purkey et al., 2018). Accordingly, in mice at ~8 weeks of age 1 × 100 Hz, HFS induces CP-AMPAR-dependent LTP in field recordings but 2 × 100 Hz induces LTP that is insensitive to CP-AMPAR antagonists (Gray et al., 2007; Lu et al., 2007). Thus, one must consider developmental age, recording method, and induction protocols. The early controversy between Plant et al. (2006) which observed CP-AMPAR recruitment following LTP induction, and Adesnik and Nicoll (2007), which did not, might be explained by the relatively small differences in induction protocol; while both studies used mice ~2–3 weeks of age and whole-cell recording pairing protocols to induce LTP, Plant et al. (2006) used slightly weaker, briefer pairing protocols on average than Adesnik and Nicoll (2007; Table 1).

However, the question still remains why might relatively weaker induction of LTP recruit CP-AMPARs while stronger induction does not? Even when CP-AMPARs are recruited to CA1 synapses after LTP (or LTD) induction, the presence of these receptors in the synapse is transient, with their own activity triggering subsequent removal (Plant et al., 2006; Sanderson et al., 2016). Thus, it is possible that when LTP is induced by stronger and/or more prolonged induction protocols, the greater resulting NMDAR Ca^2+^ influx (with no need for any Ca^2+^ contributed by CP-AMPARs) rapidly triggers these CP-AMPAR removal mechanisms to prevent even transient recruitment to the synapse. As discussed in more detail below, the CP-AMPARs transiently recruited to CA1 synapses during LTD are rapidly removed by AKAP79/150-anchored CaN signaling that promotes GluA1 S845 dephosphorylation (Sanderson et al., 2012, 2016); however, it remains to be seen whether activation of this AKAP-CaN pathway also prevents CP-AMPAR recruitment in response to strong, prolonged LTP induction stimuli.

A further complication exists whereby many studies have tried to understand AMPAR regulatory mechanisms in plasticity by manipulating the receptor itself with subunit-specific knock-outs (KO) and mutations. But there are clear problems with the “receptor-centric” approach to understanding AMPAR subunit-specific regulatory mechanism because AMPAR activity is the measurement that is used to determine synaptic strength, thus when manipulations are made to the receptor itself (*via* knockout, point mutation, deletions, etc.) it can complicate interpretations, as the manipulations can fundamentally impact receptor function even basally. Whole receptor subunit knockouts are further complicated due to compensation by other receptor subunits, and even when combined with rescue approaches can potentially produce non-physiological receptors and signaling conditions. Therefore, while there is strong evidence to suggest the involvement of CP-AMPARs in LTP, there still remains controversy and questions about the precise forms of plasticity involved and signaling mechanisms implicated. As discussed more below, a strong picture is now emerging that CP-AMPAR recruitment to synapses is heavily controlled by postsynaptic PKA signaling through GluA1 S845 phosphorylation.

### AMPAR LTP Models: Exocytosis and Lateral Diffusion

Despite the controversy of the involvement of CP-AMPARs in LTP, it is widely accepted that AMPARs are recruited to the synapse in order to increase synaptic strength. A number of non-mutually exclusive mechanisms have been proposed to explain how AMPARs get retained/recruited to the PSD in an activity-dependent manner. The overall AMPAR insertion model of LTP includes both AMPAR trafficking to the extrasynaptic plasma membrane from intracellular stores and lateral diffusion and trapping in the synapse as key mechanisms (Passafaro et al., 2001; Kennedy and Ehlers, 2006; Ehlers, 2007; Petrini et al., 2009; Opazo and Choquet, 2011; Penn et al., 2017). The primary proposed mechanism for regulated AMPAR delivery to the extrasynaptic plasma membrane is through activity-triggered exocytosis from internal stores. A seminal contribution to elucidating this plasticity mechanism was the discovery that dynamic postsynaptic membrane trafficking is required for the expression of LTP (Lledo et al., 1998; Lüscher et al., 1999; Lu et al., 2001). In addition, LTP induction acutely increases exocytosis of GluA1 AMPARs at the extrasynaptic plasma membrane (Kopec et al., 2006; Yudowski et al., 2007; Lin et al., 2009; Kennedy et al., 2010; Patterson et al., 2010; Hiester et al., 2017) that can then laterally diffuse into the PSD and be captured (Borgdorff and Choquet, 2002; Opazo and Choquet, 2011; Opazo et al., 2012; Penn et al., 2017). This AMPAR trapping is thought to involve the retention of AMPARs in “slots” in the PSD where they are optimally positioned to respond to release of glutamate from the presynaptic terminal (MacGillavry et al., 2013; Nair et al., 2013; Tang et al., 2016; Sinnen et al., 2017). In this PSD slot model, CaMKII acts on structural regulatory proteins in the PSD to create additional AMPAR slots during LTP (Araki et al., 2015; Herring and Nicoll, 2016; Walkup et al., 2016; Zeng et al., 2016, 2019), possibly by reorganizing the PSD *via* liquid-liquid phase transition, which then can effectively trap the highly mobile AMPARs through additional CaMKII phosphorylation of TARPs that increases the affinity of AMPAR-TARP complexes for the underlying synaptic architecture (Figure 3; Tomita et al., 2005; Opazo et al., 2010, 2012; Park et al., 2016). Thus, AMPAR mobilization from internal stores followed by lateral diffusion and synaptic trapping likely cooperate to increase synaptic strength during LTP.

Accordingly, most models of LTP now include the requirement for an extrasynaptic plasma membrane reserve pool of surface receptors that move laterally into the PSD to support LTP expression, with receptors residing in internal stores then being recruited to the plasma membrane to replenish this extrasynaptic reserve pool (Opazo and Choquet, 2011; Granger et al., 2013; Nicoll and Roche, 2013). Indeed, a recent article from the Choquet laboratory showed that preventing lateral mobility of AMPARs blocks the initial potentiation observed after LTP induction while blocking exocytosis from internal stores decreases potentiation only at later time points (Penn et al., 2017). One prominent pool of internal AMPARs resides in the recycling endosome (RE). REs have been observed in dendritic spines (Kennedy et al., 2010; Hiester et al., 2017) and in the dendrite shaft near the bases of spines (Park et al., 2006; Kelly et al., 2011), and it has been demonstrated that LTP relies on AMPARs that are supplied by recycling through REs (Petrini et al., 2009). Additional evidence for a requirement for receptor delivery from REs during LTP includes findings that RE trafficking proteins, including Rab11, and the vesicular fusion machinery, including multiple SNARE proteins that regulate exocytosis, are all required for LTP expression (Lledo et al., 1998; Park et al., 2004, 2006; Kennedy et al., 2010; Ahmad et al., 2012; Jurado et al., 2013; Wu et al., 2017). In addition, NMDAR activity during LTP can influence postsynaptic RE dynamics to increase recycling exocytosis (Kennedy et al., 2010; Keith et al., 2012; Woolfrey et al., 2015; Hiester et al., 2017), promote RE translocation into spines (Park et al., 2006), and increase GluA1 exocytosis (Kopec et al., 2006; Yudowski et al., 2007; Lin et al., 2009; Kennedy et al., 2010; Patterson et al., 2010; Hiester et al., 2017). As mentioned above, multiple lines of evidence suggest that when GluA2-lacking CP-AMPARs are recruited to synapses following LTP, this recruitment is transient and they are quickly replaced with GluA2-containing CI-AMPARs (McCormack et al., 2006; Plant et al., 2006; Shepherd and Huganir, 2007; Kessels and Malinow, 2009). Accordingly, PICK1 associated with GluA2 and seems to be involved in the regulated recycling/endocytosis of GluA2-containing receptors during LTP and promoting GluA1 CP-AMPAR insertion (Jaafari et al., 2012). Importantly, both PICK1 and GRIP1 are known to localize to REs (Jaafari et al., 2012; Thomas et al., 2012).

### AMPAR Regulation by Phosphorylation During LTP

Regardless of the extent to which AMPARs are recruited to the synapse from internal vs. extrasynaptic pools, there still exists the fundamental question of what signals mobilize AMPARs from these pools to the synapse? One mechanism regulating AMPAR plasma membrane insertion and synaptic recruitment is phosphorylation. In the late 1980s, it was demonstrated that kinase activity was required for the induction of LTP (Malenka et al., 1989; Malinow et al., 1989; Wyllie and Nicoll, 1994). This quickly led to a hypothesis that AMPAR subunits were phosphorylated during LTP to increase synaptic currents (Swope et al., 1992; Soderling, 1993). Since then, studies of activity-dependent AMPAR phosphorylation have focused on modification of GluA1 and GluA2 subunits, as the phosphorylation sites on these subunits were shown to be regulated by neuronal activity (Shepherd and Huganir, 2007; Lu and Roche, 2012). Strong evidence supporting the importance of phosphorylation control of AMPARs in plasticity was shown in the late 1990s, with increased GluA1 phosphorylation correlated with LTP and decreased GluA1 phosphorylation with LTD (Figures 2A,B; Barria et al., 1997; Kameyama et al., 1998; Lee et al., 1998, 2000). GluA1 phosphorylation at S831 by CaMKII and/or PKC has been shown to increase channel conductance (Derkach et al., 2007; Kristensen et al., 2011). PKA-dependent phosphorylation of S845 increases mean open time (Banke et al., 2000) and also promotes plasma membrane insertion of the receptor, especially extrasynaptically, to make GluA1 AMPARs available for subsequent synaptic recruitment during LTP (Sun et al., 2005; Oh et al., 2006; Man et al., 2007; Yang et al., 2008; He et al., 2009; Yang et al., 2010). In addition, PKC phosphorylation of GluA1 S818 increases single-channel conductance and also promotes AMPAR plasma membrane insertion, working in concert with S845 and S831 (Boehm et al., 2006; Lin et al., 2009; Jenkins et al., 2014). Recently, it was discovered that cAMP-PKA signaling can also recruit GluA3-containing receptors to synapses to increase synaptic strength, although the specific phosphorylation targets of PKA involved in this mechanism remain to be determined (Renner et al., 2017).

To study the subunit-specific requirements of LTP, many labs have used knockout, knock-in or molecular replacement approaches (Table 2). Through a combination of *in vitro* studies in organotypic slices and *ex vivo* studies in acute slices from mutant mice the three GluA1 phosphorylation sites (S818, S831, and S845) have each been shown to contribute to CA1 LTP either in combination or separately depending on the experimental conditions (Esteban et al., 2003; Lee et al., 2003, 2010; Boehm et al., 2006; Qian et al., 2012). However, it is not surprising that, as in the CP-AMPAR literature, the role of GluA1 and subunit specificity in plasticity is contentious. In particular, no manipulation that blocks phosphorylation of the AMPAR CTD residues completely blocks LTP under all conditions. For example, TBS induced LTP is normal in juvenile GluA1 S831/845A double mutant mice but is strongly impaired in adults (Lee et al., 2003). Similarly, LTP is only impaired in adult but not juvenile GluA1 knockout mice (Zamanillo et al., 1999; Jensen et al., 2003; Kolleker et al., 2003). Yet, the S845A and S831A single mutant mice show normal hippocampal LTP at all ages (Lee et al., 2010). However, young adult S845A mice are deficient in prolonged theta-train (PTT) induction of LTP that depends on CP-AMPARs, activation of postsynaptic PKA signaling by β_2_-adrenergic receptors, and PKA phosphorylation-mediated enhancement of L-type voltage-gated Ca^2+^ channels (Qian et al., 2012, 2017). Importantly, for most conditions where GluA1 KO and GluA1 phosphorylation-deficient mutant mice exhibited LTP deficits, there is evidence from other studies using the same or similar conditions (discussed in more detail below) that PKA signaling and CP-AMPARs are also required (Lu et al., 2007; Qian et al., 2012; Zhang et al., 2013; Sanderson et al., 2016).

**Table 2:** AMPAR studies in transgenic mice.

Reference(s)	Mutation	Age	Result
Kim et al. (2005; PDZ ligand)	KI mutant mice lacking the last 7 a.a. GluA1; male	3 weeks–7 months	Unaffected: Basal localization and transmission, LTP (Fields: 1 TBS, whole-cell pairing: 2 Hz, 200 pulses at 0 mV) and LTD (Fields: 1 Hz, 900 pulses, whole-cell pairing: 0.5–1 Hz, 200–300 pulses at −40 mV).
Granger et al. (2013) and Granger and Nicoll (2014a)	*Gria1–3^fl/fl^*; replaced with different mutant receptors	P17–20	No single portion of the GluA1 C-terminal tail is required for LTP (2 Hz, 90 s at 0 mV), GluA2, GluA2(Q) or GluK1 replacement sufficient to rescue LTP. GluA1 and GluA2 conditional knockouts have normal LTD (1 Hz, 15 min), GluK1 replacement in GluA1–3 conditional knockout sufficient to rescue LTD.
Zamanillo et al. (1999), Hoffman et al. (2002), Reisel et al. (2002) and Jensen et al. (2003)	GluA1 knockout	3 months, P14–42, P41–56, Adult	LTP (Fields: 1 × 100 Hz, 1 s): impaired; normal spatial learning in Morris Water Maze; LTP (Fields: 1 × 100 Hz, 1 s/Whole-cell 0.67 Hz, 3 min at 0 mV): modest/normal amount of LTP at P14 disappears by P42; LTP (TBS): decreased initially but normalizes to WT after 25 min; Normal spatial memory; spatial working memory deficits.
Meng et al. (2003)	GluA3 knockout	2–3 weeks, 2–3 months	Normal basal transmission and pre-synaptic function; LTD (1 Hz, 15 min) 12–16 days: normal; Depotentiation 2–3 weeks: normal; Enhanced LTP (100 Hz, 1 s) in adults and enhanced level of LTP saturation (6 trains of 100 Hz, 1 s with 5 min interval) in adults.
Jia et al. (1996), Gerlai et al. (1998) and Meng et al. (2003)	GluA2 knockout	P16–30, 5–8 weeks, 2–3 weeks, 2–3 months	LTP (Fields: 5 × 100 Hz, 200 ms pulses): enhanced; growth retardation and motor deficits, normal brain anatomy, increased excitability, alterations in a number of behaviors across multiple brain areas; LTD (Fields: 1 Hz, 15 min): normal; Depotentiation (HFS 100 Hz 1 s followed by LFS 1 Hz, 15 min): impaired depotentiation but enhanced LTP (100 Hz, 1 s) in adults.
Meng et al. (2003)	GluA2/3 double knockout	2-3 weeks, 2-3 months	Reduced basal transmission in adults; Normal PPR in adults; Enhanced LTD and de-depression (12–16 days); Enhanced LTP and de-potentiation (2–3 weeks old); Enhanced LTP in adult mice.
Lee et al. (2003)	GluA1 S831/845A knock-in	Young (P21–P28) and old (3 months or older)	Normal basal transmission; LTP (Fields TBS) old mostly blocked, young normal; LTD (Fields: old PP 1 Hz, 15 min and young 1 Hz, 15 min): blocked likely due to lack of receptor internalization; MWM: learning normal, impaired retention of spatial memory (delayed sessions).
Lee et al. (2010)	GluA1 S831A knock-in	Young (3 weeks) and old (3 months+)	Young-Normal basal transmission; LTP (Fields: 4 × TBS) normal; LTD: (Fields: 1 Hz) slight decrease but not statistically significant. Old-Normal basal transmission; LTP: (Fields: 4 × TBS and 1 × TBS) normal; LTD: (Fields: PP-1 Hz) normal. Normal de-potentiation and de-depression.
Lee et al. (2010) and Qian et al. (2012)	GluA1 S845A knock-in	Young (3 weeks) and old (3 months+), 6–8 weeks	Young mice have normal basal transmission and normal LTP (Fields: 4 × TBS) but virtually absent LTD (Fields: 1 Hz). Old mice have normal basal transmission and normal LTP (Fields: 4× TBS and 1 × TBS) but mostly blocked LTD (Fields: PP 1 Hz) and normal de-potentiation. At 6–8 weeks, PTT-LTP (5 Hz, 3 min in presence of β-adrenergic receptor agonist) is impaired.
Zhou et al. (2018)	GluA1 and GluA2 C-terminal tail swap knock-ins	3–4 weeks for LTP; 13–15 days for LTD	Both show normal basal transmission GluA1-C2KI has normal NMDAR LTD, impaired LTP (1 × 100 Hz, 4 × 100 Hz); GluA2-C1KI has normal mGluR LTD (100 μM (RS)-3,5-DHPG for 10 min), no NMDAR LTD (900 pulses at 1 Hz), enhanced LTP (4 × 100 Hz). With the double replacement, LTP and LTD are normal. Behavior: GluA1-C2KI impaired spatial learning and memory, GluA2-C1KI impaired contextual fear memory.

Interestingly, a recent study examining the requirement of the CTDs of GluA1 and GluA2 using chimeric knock-in mice, showed that replacing the GluA1 CTD with that of GluA2 blocks LTP, but this deficit can be rescued by reintroducing the GluA1 CTD fused to GluA2 (Zhou et al., 2018). Thus, this study reinforces that the GluA1 CTD is somehow essential for AMPAR trafficking and LTP expression, although the involvement of GluA1 CP-AMPARs was not specifically addressed. In contrast, an earlier study using KO and molecular replacement approaches reached very different conclusions; normal LTP was observed with a GluA1 construct lacking the entire C-terminal tail or by an unedited GluA2-Q construct when expressed on a conditional AMPAR GluA1–3 triple knockout background (Granger et al., 2013). However, these studies in many cases used varying protocols for inducing LTP and in some cases also used different developmental ages, which as discussed both above and below could contribute to differences in results and conclusions (Table 2). Nevertheless, a consistent result of many studies is that knocking out GluA1 results in strongly reduced extrasynaptic AMPAR surface expression and impaired LTP (Zamanillo et al., 1999; Granger et al., 2013), but knocking out GluA2 and GluA3 results in normal extrasynaptic AMPAR surface expression and LTP (Meng et al., 2003). Taken together, these findings indicate that under most circumstances GluA1 subunit trafficking maintains the reserve pool of AMPARs necessary to support LTP, and GluA1 CTD phosphorylation appears to play a crucial but complicated role in regulating this process, in part through controlling whether GluA1 homomeric CP-AMPARs contribute to this pool and can be recruited to the plasma membrane and synapse during LTP.

### AMPAR Regulation by Phosphorylation During LTD

There are multiple protocols for experimentally inducing NMDAR-dependent LTD of AMPAR synaptic strength, such as low-frequency stimulation (LFS), spike-timing-dependent plasticity (STDP) and chemical LTD with bath NMDA application (cLTD; Kameyama et al., 1998). Apart from NMDAR-dependent LTD, another mechanism for LTD induction is through a mGluR-dependent pathway. This mGluR-LTD can be induced with similar activation patterns as NMDAR-LTD, such as paired-pulse LFS (Massey and Bashir, 2007), or the group I mGluR agonist dihydroxyphenylglycine (DHPG; Palmer et al., 1997). This mGluR-dependent form of LTD will not be further discussed, and any use of LTD hereafter will be referring to NMDAR-dependent LTD. NMDAR-dependent LTD requires postsynaptic Ca^2+^ and phosphatase activity as supported by evidence that LTD expression is blocked by AP5, intracellular BAPTA (Ca^2+^ chelator; Mulkey and Malenka, 1992) and CaN or protein phosphatase 1 (PP1) inhibitors (Mulkey et al., 1994). Low-level Ca^2+^ influx from NMDARs or even basal postsynaptic Ca^2+^ levels of ~100 nM have been shown to be sufficient to support CaN and PP1 phosphatase signaling that are required for LTD (Lisman, 1989; Mulkey et al., 1993; Malenka and Bear, 2004), along with a more recently identified NMDAR conformational signaling to the kinase p38 Mitogen-Activated Kinase (MAPK; Nabavi et al., 2013; Stein et al., 2015).

Among the targets of CaN and PP1 phosphatase activity implicated in LTD is GluA1 S845; AMPARs are dephosphorylated at S845 during LTD to promote receptor endocytosis and degradation (Figure 2B; Lee et al., 1998, 2003, 2010; Fernández-Monreal et al., 2012; Sanderson et al., 2016). Endocytic zones have been discovered at the periphery of excitatory synapses (Blanpied et al., 2002) and these zones are the sites of clathrin-coated pit formation (Spacek and Harris, 1997) and AMPAR internalization (Rácz et al., 2004). Using a cLTD treatment, it was discovered that there is rapid AMPAR endocytosis (Carroll et al., 1999a,b; Beattie et al., 2000; Ehlers, 2000). It was also observed that there is decreased synaptic AMPAR content with *in vivo* LTD induction (Heynen et al., 2000). For NMDAR-dependent AMPAR internalization (like LTD) Ca^2+^ influx and activation of CaN are needed (Beattie et al., 2000; Ehlers, 2000; Zhou et al., 2001). Interestingly, as mentioned above it was recently identified that transient incorporation of CP-AMPARs also occurs during LTD induction downstream of PKA signaling, but these receptors are rapidly removed before the end of the induction stimulus by CaN signaling (Sanderson et al., 2016). This transient CP-AMPAR recruitment is reminiscent of some forms of LTP discussed above; however, the time-scale of removal of the recently recruited CP-AMPAR is different (removal within ~15–30 min post-LTP induction vs. within 6–15 min during LTD induction).

**Figure 4:**
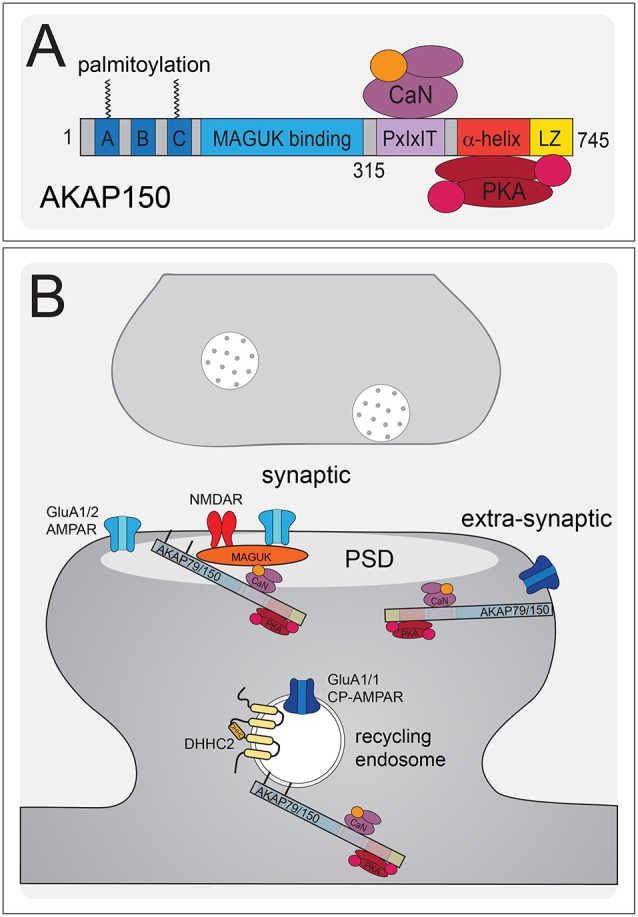
AKAP79/150 localizes bidirectional PKA-CaN signaling to key postsynaptic signaling nodes.** (A)** Schematic of AKAP79/150 highlighting the C-terminal PKA and CaN signaling protein binding partners and anchoring domains, the internal MAGUK binding domain, and the N-terminal polybasic membrane targeting domains (A–C) containing two sites of S-palmitoylation. **(B)** AKAP79/150 is targeted to the PSD, extrasynaptic membrane, and recycling endosome (RE) through protein-protein and membrane lipid interactions that are modulated by S-palmitoylation within the N-terminal polybasic domains. AKAP79/150 anchors the phosphatase CaN and kinase PKA to provide bidirectional signaling in control of AMPARs.

**Figure 5:**
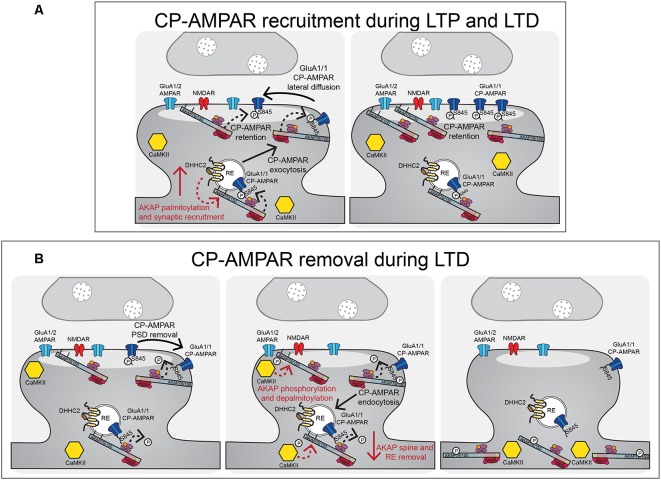
AKAP79/150-anchored PKA and CaN control CP-AMPAR trafficking during LTP and LTD. **(A)** During LTP and LTD, AKAP79/150 is recruited to dendritic spines and recycling endosomes through palmitoylation by DHHC2. AKAP-anchored PKA phosphorylates GluA1 at S845 to promote CP-AMPAR synaptic recruitment during both LTP and LTD. **(B)** During LTD, AKAP-anchored CaN then dephosphorylates GluA1 at S845 resulting in CP-AMPAR removal from the synapse and endocytosis. AKAP79/150 itself is then subsequently removed from spines and recycling endosomes to prevent rephosphorylation of GluA1 by PKA. This AKAP79/150 translocation from the synapse is downstream of CaN-dependent F-actin reorganization and AKAP depalmitoylation that is promoted by CaMKII mediated in part by through phosphorylation of the N-terminal targeting domain.

Though it is widely accepted that AMPARs are removed during LTD, there is no coherent mechanistic model. The activities of PKA, CaMKII, Cyclic Dependent Kinase 5 (CDK5), p38MAPK, and Glycogen Synthase Kinase (GSK3) have all been implicated in LTD (Collingridge et al., 2010; Coultrap et al., 2014). The CTD of GluA2 is phosphorylated at S880 in the PDZ ligand to inhibit GRIP/ABP binding and promote PICK1 binding and disrupting scaffolding interactions with this PDZ ligand can block LTD (Daw et al., 2000; Kim et al., 2001; Seidenman et al., 2003). Accordingly, experiments using GluA1 and GluA2 CTD chimera mice found a requirement for GluA2 CTD but not the GluA1 CTD in LTD (Park et al., 2016). Nonetheless, both GluA2 and GluA2/3 double knockout retain LTD (Meng et al., 2003). In addition, similar to LTP above, single-subunit replacement approaches in a GluA1–3 triple conditional knockout background found that either GluA1 or unedited GluA2-Q alone can support LTD (Granger and Nicoll, 2014b). However, while LTD is normal in complete GluA1 knock-out (Selcher et al., 2012), it is impaired in the GluA1 S845A mutant (Lee et al., 2010). Thus, like AMPAR recruitment in LTP, these studies together indicate that the mechanisms controlling AMPAR removal during LTD can act through multiple subunits. However, none of the above studies specifically examined a role for CP-AMPARs, although the finding that the GluA1 S845A mutant, which lacks extrasynaptic GluA1 homomers in the hippocampus, exhibits impaired LTD suggested their possible involvement (He et al., 2009). This possible involvement of CP-AMPARs in LTD was later confirmed in studies discussed more below characterizing how PKA and CaN signaling organized by the postsynaptic scaffold protein AKAP79/150 regulates CP-AMPARs in LTD as well as LTP (Figures 4, 5).

## Regulation of CP-AMPAR-Mediated Plasticity by AKAP79/150-Anchored PKA and CaN

Regardless of these remaining mechanistic questions regarding GluA1 vs. GluA2 involvement in LTP and LTD, emerging evidence indicates that many of the kinases and phosphatases that regulate GluA1 phosphorylation and AMPAR trafficking, including CaMKII (Thiagarajan et al., 2002, 2005; Groth et al., 2011), PKA (Goel et al., 2011; Diering et al., 2014), and CaN (Kim and Ziff, 2014), play key roles regulating CP-AMPARs to impact LTP, LTD, and homeostatic plasticity (Thiagarajan et al., 2005; Plant et al., 2006; Lu et al., 2007; Yang et al., 2010; Goel et al., 2011; Soares et al., 2013; Kim and Ziff, 2014; Kim et al., 2015; Megill et al., 2015; Woolfrey and Dell’Acqua, 2015; Sanderson et al., 2016). By creating knock-in mice to disrupt PKA (D36, ΔPKA) and CaN (ΔPIX) anchoring to the postsynaptic scaffold protein AKAP79/150 (79 human/150 rodent; *Akap5* gene; Figure 4A; Table 3), we and others found that AKAP-PKA/CaN signaling bi-directionally regulates GluA1-S845 phosphorylation to control the balance of CP-AMPAR recruitment/removal at CA1 synapses basally and during LTP/LTD (Figure 5; Lu et al., 2007; Sanderson et al., 2012, 2016; Zhang et al., 2013). Because of four S845 phosphorylation sites in a GluA1 homomer (compared to two in a GluA1/2 heteromer), this PKA/CaN metaplasticity appears to be especially key for CP-AMPAR regulation.

**Table 3:** AKAP150 transgenic mouse model studies.

AKAP150 mutation	Phenotype	References
Knockout (two different lines)	**Basal**	Tunquist et al. (2008)
	2 weeks normal or slightly enhanced
	8 weeks normal
	**LTP (100 Hz, 1 s)**
	8 weeks normal
	**LTD (1 Hz, 15 min)**
	2 weeks normal
	8–16 weeks impaired (NMDAR vs. mGluR? Not determined)	Weisenhaus et al. (2010)
	**Behavior**
	Modest deficits in spatial memory
	Normal reversal learning
	Impaired cerebellar behaviors
	Reduced pilocarpine seizures
	**PTT-LTP (5 Hz, 3 min in presence of a β-adrenergic receptor agonist)** CP-AMPAR dependent, 6–8 weeks impaired	Zhang et al. (2013)
D36 (PKA anchoring-deficient)	**Basal**	Lu et al. (2007)
	Normal
	**LTP (100 Hz, 1 s)**
	CP-AMPAR and PKA independent, 4–5 weeks normal	Lu et al. (2008)
	CP-AMPAR and PKA dependent, 8 weeks impaired
	**LTD (1 Hz, 15 min)**	Weisenhaus et al. (2010)
	2 weeks, impaired (retain ~10%)
	**Depotentiation (100 Hz, 1 s and 5 min later 1 Hz, 15 min)**
	Normal
	**Behavior**
	Impaired reversal learning
	Normal spatial learning, working memory, and open field behaviors
	**PTT-LTP (5 Hz, 3 min in presence of a β-adrenergic receptor agonist)** 6–8 weeks partially impaired	Zhang et al. (2013)
ΔPKA (PKA anchoring-deficient)	**Basal**	Sanderson et al. (2016)
	Normal
	**LTP (100 Hz, 1 s)**
	2 weeks normal magnitude (but unlike WT is not CP-AMPAR dependent)
	**LTD (1 Hz, 15 min)**
	CP-AMPAR dependent, 2 weeks impaired (retain ~10%)
ΔPIX (CaN anchoring-deficient)	**Basal**	Sanderson et al. (2012)
	Normal but increased CP-AMPARs
	**LTP (100 Hz, 1 s)**
	2–3 weeks enhanced due to increased CP-AMPAR synaptic recruitment, but 50 Hz, 2 s normal
	**Depotentiation (100 Hz, 1 s and 30 min later 1 Hz, 15 min)**
	Impaired: de-potentiates to a similar amount but does not reach WT baseline levels
	**LTD (Fields 1 Hz, 15 min or Whole-cell 1 Hz, 6 min paired at −30 mV)**
	2 weeks impaired due to decreased CP-AMPAR synaptic removal (1 Hz PP 900 pulses, 50 ms interval LTD and 10 Hz transient depression also impaired)
CS (palmitoylation-deficient)	**Basal**	Purkey et al. (2018)
	Normal but increased CP-AMPARs
	**LTP (Fields 100 Hz, 1 s)**
	2–3 weeks impaired
	**LTP (Whole-cell 2 × 100 Hz, 0 mV)**
	CP-AMPAR dependent, 2–3 weeks impaired
	**LTP (Whole-cell 3 Hz, 90 s, 0 mV)**
	CP-AMPAR independent, 2–3 weeks normal
	**LTD (Fields 1 Hz, 15 min)**
	2 weeks normal
	**De-depression (Fields 1 Hz LTD, 15 min and 15 min later 100 Hz, 1 s LTP)**
	CP-AMPAR dependent, 2 weeks enhanced

PKA phosphorylation of S845 has also been linked to CP-AMPAR synaptic incorporation during homeostatic scaling-up in cultured cortical neurons (Kim and Ziff, 2014; Diering et al., 2014) and in visual cortex in response to light deprivation (Goel et al., 2011), with phospho-deficient S845A knock-in mice exhibiting impaired scaling-up in both systems. Yet inhibition of CaN, which also occurs during neuronal silencing due to decreased Ca^2+^, is sufficient to increase S845 phosphorylation and induce scaling-up through CP-AMPARs in cortical neurons (Kim and Ziff, 2014). Thus, it may not be the absolute levels but the *balance* of PKA vs. CaN signaling that exerts metaplastic control over not only LTP/LTD but also homeostatic plasticity. Consistent with this idea, using hippocampal neurons cultured from AKAP150 ΔPKA and ΔPIX knock-in mice, we recently demonstrated that AKAP-anchored PKA and CaN also oppose each other to control S845 phosphorylation and CP-AMPAR incorporation during homeostatic scaling-up *in vitro* in hippocampal neuron cultures (Sanderson et al., 2018).

AKAP79/150 is highly enriched in the hippocampus at the PSD with AMPARs (Carr et al., 1992b; Gomez et al., 2002; Lu et al., 2007; Li et al., 2012), in REs (Keith et al., 2012; Woolfrey et al., 2015; Purkey et al., 2018), and in the extrasynaptic plasma membrane (Dell’Acqua et al., 1998). AKAP79/150 is known to bind the kinase PKA (Carr et al., 1992a,b) at the distal C-terminus of the scaffold using a canonical amphipathic α-helix that is also found in other AKAP family members (Figure 4A). However, unlike most other AKAPs, AKAP79/150 can also bind the CaN phosphatase catalytic A subunit through a PxIxIT-type docking motif located just N-terminal to the PKA binding site (Coghlan et al., 1995; Dell’Acqua et al., 2002; Oliveria et al., 2007, 2012; Li et al., 2012). Finally, AKAP79/150 interacts with PKC (Klauck et al., 1996; Faux et al., 1999), which is activated by Ca^2+^ and diacylglycerol (DAG), near the N-terminus through an inhibitory pseudo-substrate-like motif that is regulated by Ca^2+^-calmodulin binding (Faux and Scott, 1997). This multivalent scaffolding is particularly important when considering the synaptic signaling that requires bidirectional kinase and phosphatase signaling to control the phosphorylation state of AMPARs and other synaptic proteins during plasticity.

The N-terminus of the AKAP79/150 protein participates in many different cellular activities in addition to PKC anchoring (Klauck et al., 1996; Dell’Acqua et al., 1998; Gomez et al., 2002; Gorski et al., 2005; Tavalin, 2008), including most importantly targeting to the plasma membrane. Immunocytochemistry for AKAP150 in hippocampal neurons shows a clear association with the somatodendritic plasma membrane with notable enrichment in dendritic spines. Which begs the question: how is AKAP79/150 itself targeted to the synapses? Previous studies showed that within the N-terminus exist three membrane targeting polybasic domains (A, B, and C; Dell’Acqua et al., 1998), two of which also contain conserved palmitoylation sites that will be discussed further below (Delint-Ramirez et al., 2011; Keith et al., 2012; Woolfrey and Dell’Acqua, 2015; Woolfrey et al., 2018). AKAP79/150 interacts with the plasma membrane directly through electrostatic interactions of the three polybasic domains with the acidic phospholipid phosphatidylinositol 4,5-bisphosphate (PIP_2_; Dell’Acqua et al., 1998). AKAP79/150 can also bind N-cadherin (a transsynaptic cell adhesion molecule) and the actin cytoskeleton (F-actin) *via* these domains (Dell’Acqua et al., 2002; Gomez et al., 2002; Gorski et al., 2005). AKAP79/150 is further targeted to postsynaptic glutamate receptor signaling complexes in the PSD through its internal MAGUK binding domain (Colledge et al., 2000; Bhattacharyya et al., 2009; Nikandrova et al., 2010). The MAGUK family of proteins, specifically PSD-95 and SAP97 (Colledge et al., 2000; Robertson et al., 2009), interact with AKAP79/150 by way of their C-terminal SH3 and GK domains (Colledge et al., 2000) and these interactions allow assembly of large signaling complexes by bringing the AKAP near AMPARs and NMDARs in the PSD as well as other subcellular compartments (discussed more below). Accordingly, AKAP79/150 can control synaptic AMPAR content both through acting as structural protein and through anchored PKA and CaN signaling (Robertson et al., 2009; Sanderson et al., 2012, 2016).

The first evidence of AKAP-anchored PKA influencing AMPAR-mediated transmission came from pharmacological studies utilizing a peptide Ht31 that interferes with AKAP-PKA binding (Carr et al., 1992a), revealing that blocking this interaction resulted in decreased synaptic and extrasynaptic AMPAR currents (Rosenmund et al., 1994). Later studies found that AKAP79/150 is the primary AKAP targeting PKA to postsynaptic spines and the PSD and that AKAP-anchored CaN signaling was responsible for the decreased AMPAR activity observed when PKA anchoring was disrupted (Dell’Acqua et al., 2002; Tavalin et al., 2002; Hoshi et al., 2005). AKAP79/150 can interact indirectly with GluA1-AMPARs *via* SAP97 and also *via* PSD-95 and TARPs (Colledge et al., 2000; Tavalin et al., 2002; Bhattacharyya et al., 2009). Importantly, several studies have also shown that AKAP79/150-anchoring of PKA promotes phosphorylation of S845 on GluA1 to impact the regulation of LTP, LTD, and homeostatic plasticity by CP-AMPARs (Lu et al., 2007, 2008; Tunquist et al., 2008; Weisenhaus et al., 2010; Zhang et al., 2013; Diering et al., 2014; Sanderson et al., 2016, 2018). Additional studies in heterologous systems found that assembly and trafficking of CP-AMPARs can be further regulated by AKAP-anchored PKC through phosphorylation of GluA1 S831; however, it remains to be determined whether these PKC mechanisms also operate *in vivo* to control plasticity at CA1 synapses (Tavalin, 2008; Summers et al., 2019). Interestingly, as discussed in more detail below, during both LTP and LTD, AKAP79/150 facilitates CP-AMPAR recruitment to and removal from CA1 synapses *via* its anchoring of PKA and CaN, respectively (Bhattacharyya et al., 2009; Jurado et al., 2010; Sanderson et al., 2012, 2016, 2018). In line with their clear importance in controlling neuronal functions, AKAP79/150 and other AKAPs have been implicated in diseases such as seizures, addiction, pain, and neurodegeneration like AD and Parkinson’s disease (Wild and Dell’Acqua, 2018).

A number of mutant mouse models have been used to understand the functional implications of manipulating AKAP79/150 anchoring at the synapse (Table 3). As explained below, the AKAP150 total knockout in general exhibits surprisingly mild phenotypes, especially with respect to synaptic function given the deletion of such an important signaling hub. It is a notable caveat that compensation can occur especially when knocking out a protein from birth. For example, other AKAPs, such as AKAP250/Gravin (Havekes et al., 2012), that also anchor a similar set of signaling molecules could compensate for a total AKAP150 knockout. Further, it can be complicated figuring out what particular component of the scaffold is responsible for what phenotypic expression due to the multivalent capacity of the protein, especially considering that some of these components functionally oppose each other (i.e., PKA and CaN). So, to circumvent these issues, our laboratory and others have studied the importance of AKAP79/150 PKA and CaN anchoring in hippocampal neurons using knockdown/replacement and knock-in mutations to specifically alter the different enzyme anchoring sites (Table 3).

### AKAP150-PKA Binding Deficient Mutants ΔPKA and D36

To study AKAP150-PKA uncoupling, specific mutations that perturb AKAP-PKA binding through mutating the amphipathic α-helix that PKA-RII binds to on the AKAP were generated in two different knock-in mouse models, D36 and ΔPKA (Table 3). The D36 AKAP150 PKA-binding mutant was developed first by truncating the last 36 amino acids of the C-terminal domain of the AKAP. D36 mice were found to have normal basal excitatory transmission and S845 phosphorylation (in 2, 4–5 and 7–12 week old animals) but impaired activity-induced phosphorylation of GluA1 S845 (Lu et al., 2007, 2008). LTP was normal in ~4 week-old D36 animals when LTP induced with 1 × 100 Hz stimulation was found to be insensitive to inhibitors of PKA and CP-AMPARs, but impaired at ~8 weeks of age when this LTP was prevented by PKA and CP-AMPAR inhibitors (Lu et al., 2007). Furthermore, LTD was impaired in 2-week-old D36 animals but depotentiation of prior LTP was normal (Lu et al., 2008). These mice also exhibited impairment in the reversal-learning phase in an operant conditioning task (Weisenhaus et al., 2010). Interestingly, in parallel analyses, complete AKAP150 KO mice exhibited no alterations in LTD in juveniles, LTP in juveniles or adults, or operant learning (Weisenhaus et al., 2010). However, in another study, an independent AKAP150 KO line exhibited reduced basal GluA1 S845 phosphorylation, impaired LTD (but normal LTP) in adult animals, and mild spatial learning impairment in the Morris water maze (Table 3, Tunquist et al., 2008).

The D36 model is more specific than complete loss of all AKAP150 functions in KO animals but results in deletion of not only the PKA anchoring site but also of a modified leucine-zipper (LZ) motif that helps recruit the AKAP to L-type Ca^2+^ channel signaling complexes (Oliveria et al., 2007; Murphy et al., 2019). To circumvent any issues with deleting this LZ motif, our laboratory independently developed the PKA anchoring-deficient mutant AKAP150ΔPKA that just removes 10 amino acids (709–718) from the N-terminal portion of the amphipathic α-helix PKA-RII binding site (Table 3; Murphy et al., 2014; Sanderson et al., 2016). Overall, phenotypes for the D36 and ΔPKA animals are very similar. ΔPKA animals have normal basal CA1 synaptic transmission (both excitatory and inhibitory) at 2–3 weeks of age but decreased GluA1 S845 phosphorylation basally. Similar to D36, 2 week-old ΔPKA animals retained only ~10% of CA1 LTD expression, a deficit which was subsequently shown to be due to a failure, compared to WT, to transiently recruit CP-AMPARs to synapses during the 1 Hz induction stimulus, as assessed by both rectification measurements and use of the CP-AMPAR antagonists NASPM and IEM1460. Yet interestingly, 1 × 100 Hz LTP expression in 2 week-old ΔPKA animals was normal but insensitive to IEM1460, unlike WT LTP that was sensitive to IEM1460 at this age. Importantly, D36 and complete AKAP150 KO mice were also found to be deficient in PTT-LTP at CA1 synapses, which requires β-adrenergic-cAMP-PKA signaling and CP-AMPARs (Zhang et al., 2013). Overall, these AKAP150 KO, ΔPKA and D36 mouse studies indicate that AKAP-anchored PKA promotes GluA1 S845 phosphorylation and CP-AMPAR recruitment both during LTP and LTD (Figure 5).

### AKAP150-CaN Binding Deficient Mutant ΔPIX

To study the disruption of AKAP150-CaN anchoring, our laboratory generated a mutant mouse model that deletes seven amino acids (655-PIAIIIT-661), which we call ΔPIX, containing the CaN docking PxIxIT motif (Table 3). AKAP150ΔPIX mice at 2–3 weeks of age exhibit overall normal basal synaptic strength at CA1 synapses but with enhanced basal GluA1 S845 phosphorylation (Sanderson et al., 2012). Mice with the ΔPIX mutation at 2–3 weeks of age also exhibited impaired NMDAR-dependent LTD and enhanced 1 × 100 Hz LTP. This LTD impairment in ΔPIX mice was associated with impaired dephosphorylation of GluA-S845 and a lack of removal of GluA1 and AKAP150 from the PSD following LTD. Furthermore, ΔPIX animals showed enhanced basal activity of CP-AMPARs at CA1 synapses that acted to both inhibit LTD, due to impaired removal, and facilitate enhanced LTP, due to additional recruitment. Thus, AKAP-anchored CaN appears to be important for restricting both basal and plasticity-induced synaptic incorporation of CP-AMPARs by opposing PKA-mediated phosphorylation of S845 and is essential for dephosphorylation and the removal of CP-AMPARs that are transiently recruited to CA1 synapses during LTD (Figure 5; Sanderson et al., 2012, 2016).

### AKAP79/150 Palmitoylation and Postsynaptic Trafficking During LTP and LTD

AKAP79/150 is targeted to dendritic spines where it is present in both the PSD and extrasynaptic plasma membrane (Carr et al., 1992b; Colledge et al., 2000; Gomez et al., 2002; Tunquist et al., 2008; Weisenhaus et al., 2010), but we more recently discovered that it is also localized to dendritic REs (Keith et al., 2012; Woolfrey et al., 2015). Importantly, as discussed above, PKA/CaN regulation of AMPAR phosphorylation is thought to control recruitment/removal of synaptic AMPARs during LTP/LTD in part through coordinately regulating RE exocytosis and endocytosis at the extrasynaptic membrane to provide the reserve pool of extrasynaptic receptors available for lateral exchange in and out of the PSD (Beattie et al., 2000; Ehlers, 2000; Esteban et al., 2003; Park et al., 2004; Brown et al., 2005; Oh et al., 2006; Ehlers et al., 2007; Petrini et al., 2009; Opazo et al., 2010; Opazo and Choquet, 2011; Fernández-Monreal et al., 2012). While our previous work demonstrated AKAP79/150 targeting to the plasma membrane, in general, is mediated by binding of its three N-terminal polybasic domains (A, B, C) to acidic lipids (i.e., PI-4, 5-P_2_) and cortical F-actin (Dell’Acqua et al., 1998; Gomez et al., 2002; Horne and Dell’Acqua, 2007), our recent work found that AKAP targeting to REs requires additional S-palmitoylation on two Cys residues (C36 and C129 human/123 mouse) in this N-terminal domain (Figures 4A,B; Keith et al., 2012; Woolfrey et al., 2015).

S-palmitoylation is catalyzed by a family of DHHC palmitoyl acyltransferases (PATs) that covalently attach the C-16 fatty acid palmitate to Cys residues *via* a thioester linkage (Fukata and Fukata, 2010; Greaves and Chamberlain, 2011). In contrast to other lipidations like myristoylation and prenylation, palmitoylation is reversible, with palmitate removal being catalyzed by thioesterases. Of note, the palmitoylation levels of AKAP150 and other synaptic proteins can be affected by seizures and anticonvulsants (Kang et al., 2008; Keith et al., 2012; Kay et al., 2015), and several DHHC PATs have been linked to nervous system disorders, including Huntington’s, schizophrenia, and X-linked intellectual disability (Huang et al., 2004; Mukai et al., 2004, 2008; Mansouri et al., 2005; Fukata and Fukata, 2010). Palmitoylation frequently directs proteins to cholesterol-rich, detergent-resistant lipid-raft membrane domains (Fukata and Fukata, 2010; Greaves and Chamberlain, 2011). Notably, the PSD is biochemically defined by its detergent-insolubility and many PSD proteins are palmitoylated and show lipid-raft association (Fukata and Fukata, 2010), including the central PSD scaffold and AKAP binding partner PSD-95 (Topinka and Bredt, 1998; Craven et al., 1999; Colledge et al., 2000; Robertson et al., 2009). Accordingly, our recent work (Purkey et al., 2018) indicates that AKAP palmitoylation increases its association with the PSD and is required for CP-AMPAR synaptic incorporation during LTP (Figure 5), as discussed in more detail below.

Palmitoylation of AKAP79/150, unlike for PSD-95, is not a requirement for general plasma membrane targeting, because AKAP79/150 CS mutants that cannot be palmitoylated are still targeted to the plasma membrane. Further work by our group identified DHHC2 as the PAT responsible for AKAP79/150 palmitoylation (Woolfrey et al., 2015) and found that palmitoylation specifically targets AKAP79/150 to the RE and lipid rafts in the core PSD (Figure 4B; Delint-Ramirez et al., 2011; Keith et al., 2012; Woolfrey et al., 2015; Purkey et al., 2018). Importantly, we also found that cLTP increases and NMDA-cLTD decreases AKAP palmitoylation and spine targeting in cultured neurons (Keith et al., 2012), indicating that AKAP palmitoylation is bi-directionally regulated by neuronal activity to modulate its synaptic localization (Figure 5). Palmitoylation-mediated recruitment of additional AKAP79 to dendritic spines following cLTP was subsequently found to require DHHC2 expression (Woolfrey et al., 2015), while depalmitoylation-mediated removal of AKAP79 from dendritic spines following cLTD was found to require CaMKII activity, perhaps helping explain recent findings that CaMKII not only mediates LTP but is also required for LTD (Coultrap et al., 2014; Woolfrey et al., 2018). Interestingly, DHHC2 is specifically localized in REs and also palmitoylates PSD-95 to control its PSD clustering (Greaves et al., 2011; Fukata et al., 2013; Woolfrey et al., 2015). Yet, DHHC2 knock-down closely phenocopied that of the AKAP79CS mutant with respect to altered cLTP regulation of RE exocytosis, AKAP spine localization, and AMPAR potentiation, with these phenotypes being rescued by a constitutively lipidated/depalmitoylation resistant N-myristoylated-AKAP79 mutant (Woolfrey et al., 2015).

### AKAP150 Palmitoylation-Deficient CS Mutant Knock-In Mice

Functionally, either acute overexpression of AKAP79C36, 129S in neuronal cultures or chronic knock-in of the palmitoylation-deficient AKAP150 CS mutation in mice (Table 3) resulted in enhanced basal AMPAR transmission in hippocampal neurons measured by recording of miniature excitatory postsynaptic currents (mEPSCs; Keith et al., 2012; Purkey et al., 2018), thus indicating that AKAP palmitoylation is important for its function as an AMPAR regulator. The enhanced basal AMPAR transmission seen for the AKAP CS mutant in both culture and at CA1 synapses *ex vivo* was associated with a basal increase in synaptic CP-AMPAR activity (Keith et al., 2012; Purkey et al., 2018), which is reminiscent of CaN-anchoring deficient ΔPIX mutant (Sanderson et al., 2012, 2018). However, unlike ΔPIX mice that exhibited impaired LTD and enhanced 1 × 100 Hz HFS LTP, 2–3 week-old AKAP CS mice exhibited normal LTD and selective impairment in CP-AMPAR-dependent LTP induced with 100 Hz HFS but not CP-AMPAR-independent LTP induced with 3 Hz, 90 s 0 mV pairing (Purkey et al., 2018). Thus, in contrast to ΔPIX mice, in AKAP CS mice the basal presence of CP-AMPARs at CA1 synapses appears to be interfering with additional CP-AMPAR recruitment in response to a weaker, brief LTP induction stimulus but not their removal during LTD or their replacement with GluA2-containing AMPARs in response to a stronger, more prolonged LTP induction stimulus. Accordingly, prior induction of LTD in AKAP CS mice to remove CP-AMPARs was able to restore CP-AMPAR synaptic recruitment in response to subsequent induction of LTP/de-depression with brief 1 × 100 Hz HFS (Purkey et al., 2018). Overall, AKAP79/150 palmitoylation appears to regulate a number of important aspects of neuronal function that impact both basal transmission and activity-induced plasticity, including most notably CP-AMPAR synaptic recruitment.

## Conclusions

Overall, by avoiding complications associated with mutating the AMPARs themselves and instead focusing on manipulating upstream kinase/phosphatase regulatory mechanisms, the knock-in mouse studies described above characterizing the roles of AKAP-anchored PKA, CaN, and S-palmitoylation in LTP/LTD have provided a substantial amount of additional evidence for the importance of GluA1 S845 phosphorylation and CP-AMPARs in regulating hippocampal synaptic plasticity. In addition, other studies mentioned above have implicated similar AKAP-PKA/CaN bidirectional control of GluA1 S845 phosphorylation in regulation of CP-AMPAR synaptic recruitment during homeostatic potentiation in cultured hippocampal and cortical neurons. However, a number of important questions remain to be addressed with respect to the CP-AMPAR regulation of synaptic plasticity. In particular, we do not understand the specific route that GluA1 CP-AMPARs travel along on their way to the synapse in terms of trafficking through intracellular recycling stores and the extrasynaptic plasma membrane. While there is evidence that PKA phosphorylation of GluA1 S845 helps prevent endo-lysosomal degradation of GluA1, promotes recycling to the plasma membrane, and stabilizes CP-AMPARs in the extrasynaptic membrane (Ehlers, 2000; Oh et al., 2006; He et al., 2009; Yang et al., 2010; Fernández-Monreal et al., 2012), we still do not know where or how these GluA1 homomeric CP-AMPARs are assembled. Intriguingly, a recent single-molecule trafficking study reported that GluA1 and GluA2 subunit monomers and dimers rapidly exchange (100–200 ms) in and out of tetrameric AMPAR assemblies at the plasma membrane and laterally diffuse in and out of the synapse more readily than tetramers (Morise et al., 2019). Thus, GluA1 homomers could be rapidly assembled from monomers and dimers in response to phosphorylation during plasticity induction, either directly in the synapse or in other compartments *via* subunit exchange. In this regard, the role of AKAP79/150-anchored PKC signaling in CP-AMPAR regulation during LTP perhaps warrants additional investigation, as a recent study indicates that AKAP-PKC mediated phosphorylation of S831 can promote GluA1 homomer formation in a heterologous expression system (Summers et al., 2019). In addition, we do not know whether these AKAP-mediated regulatory mechanisms also control CP-AMPAR mediated plasticity in the hippocampus during pathophysiological states, such as during ischemia and with amyloid-beta exposure during Alzheimer’s disease (Liu and Zukin, 2007; Whitcomb et al., 2015), or in other brain regions where changes in CP-AMPAR synaptic incorporation have been observed, such as in the nucleus accumbens and ventral tegmental area in drug addiction models and in the basolateral amygdala in fear extinction learning (Clem and Huganir, 2010; Wolf and Tseng, 2012). Finally, we do not know what specific downstream signaling pathways are being engaged by CP-AMPAR synaptic Ca^2+^ influx to control LTP vs. LTD and synaptic metaplasticity in the hippocampus or these other brain regions. Thus, a great deal of interesting and potentially impactful research awaits the field in the future.

## Author Contributions

AP and MD’A wrote this review article and prepared the figures and tables.

## Conflict of Interest

The authors declare that the research was conducted in the absence of any commercial or financial relationships that could be construed as a potential conflict of interest.
